# Classification between Failed Nodes and Left Nodes in Mobile Asset Tracking Systems †

**DOI:** 10.3390/s16020240

**Published:** 2016-02-18

**Authors:** Kwangsoo Kim, Jae-Yeon Jin, Seong-il Jin

**Affiliations:** 1 UGS Convergence Research Division, ETRI, 218 Gajeong-ro, Yuseong-gu, Daejeon 34129, Korea; enoch@etri.re.kr; 2 Department of Computer Engineering, Chungnam National University, 99 Daehak-ro, Yuseong-gu, Daejeon 34134, Korea; daramkun@daram.pe.kr

**Keywords:** wireless sensor network, mobile node, medical asset, node classification, failure detection

## Abstract

Medical asset tracking systems track a medical device with a mobile node and determine its status as either in or out, because it can leave a monitoring area. Due to a failed node, this system may decide that a mobile asset is outside the area, even though it is within the area. In this paper, an efficient classification method is proposed to separate mobile nodes disconnected from a wireless sensor network between nodes with faults and a node that actually has left the monitoring region. The proposed scheme uses two trends extracted from the neighboring nodes of a disconnected mobile node. First is the trend in a series of the neighbor counts; the second is that of the ratios of the boundary nodes included in the neighbors. Based on such trends, the proposed method separates failed nodes from mobile nodes that are disconnected from a wireless sensor network without failures. The proposed method is evaluated using both real data generated from a medical asset tracking system and also using simulations with the network simulator (ns-2). The experimental results show that the proposed method correctly differentiates between failed nodes and nodes that are no longer in the monitoring region, including the cases that the conventional methods fail to detect.

## 1. Introduction

With the development of various embedded computing platforms and low power sensing components, a diverse set of applications using wireless sensor networks (WSN) has been implemented in the past decade. These applications can be divided into two categories based on the type of sensor nodes that they use. These are static sensor networks and mobile sensor networks. Static sensor networks are composed of many stationary nodes, and these sensor nodes are located at various positions to sense local phenomena around them. These nodes have the characteristic that their positions rarely change. Such systems are used in applications, such as environmental monitoring [1,2], structural health monitoring [3,4,5], industrial asset monitoring [6], building automation [7,8], static asset management [9] and traffic monitoring applications [10]. On the other hand, mobile sensor networks are composed of many mobile nodes. In these networks, one or more mobile nodes are attached to a mobile object to monitor its movement and current location in real time. Such systems can be applied to healthcare systems [11,12,13], mobile asset tracking [14,15,16,17] and human monitoring applications [18].

Among various applications where WSN devices are applied, one important application that these systems can benefit is the application of mobile asset tracking. By attaching miniature sized wireless devices, the location and presence of various high-cost mobile assets can be tracked. As an example, in the hospital environment, while patients are encouraged to access different types of devices that are provided for their convenience, the problem of burglary is a major issue. In a recent report, hospitals face yearly losses of $150,000 dollars due to such unpredicted losses of assets [19]. Currently, designated personnel monitor such assets manually, but the use of wireless systems can easily and effectively minimize such incidents.

**Figure 1:**
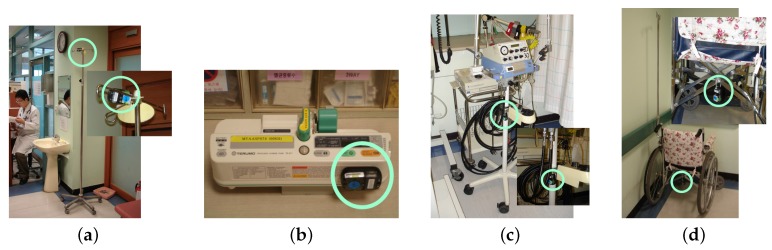
Mobile medical assets with mobile nodes. (**a**) IV Pole; (**b**) Syringe Pump; (**c**) Ventilator; (**d**) Wheel Chair [17]. (With the permission of IEEE publisher).

To provide such a wireless system, a mobile asset tracking system was developed and installed at the emergency room of a medical center with the objective of reducing nurses’ workloads. This experience also served the purpose of validating the performance of the asset tracking system in a real application environment. In the system, mobile assets (e.g., wheelchairs and syringe pumps) move within the emergency room, freely move out of the tracking area and then can choose to come back to the region, as well. A mobile node is attached to a target asset, and the system tracks the current location of the node using the characteristics of the RF (radio frequency) signals. Specifically, this system is used for two different tasks: asset discovery and asset management. For asset discovery, the approximate location of each mobile medical asset is required; on the other hand, for asset management, the accurate quantity of assets both inside and outside of the target region needs to be known. This system displays the current location of various mobile assets and classifies them as either an inside asset or an outside asset based on the assets’ current location and the connection status of the wireless node attached to the mobile asset. Each medical asset with a mobile node is shown in Figure 1.

The challenge in designing such a system is the fact that wireless nodes can fail, and it is difficult for the system to distinguish nodes that left the coverage area from the nodes that failed to provide effective service. The main reason behind this complication is the fact that these two types of activities show similar behavior: disconnection from the network. Empirically, during the initial studies with the mobile asset tracking system, several reports regarding this issue were generated. As the outcome of radio dis-connectivity from the network is the same for the two cases, the previous patterns of the wireless connection are carefully investigated to devise an efficient classification method. This method has to effectively distinguish between nodes that leave the monitoring area and faulty nodes that fail to provide service while (physically) within the monitoring area. Specifically, in this work, the classification method uses trends of the neighbor count sequences and link quality statistics for the suddenly disconnected (due to any reason) mobile nodes and also the same information from (previously connected) neighboring boundary nodes in the monitoring area. These collected trends are monitored to distinguish nodes that naturally leave the monitoring area from the nodes that fail to provide satisfactory service.

The evaluations using real traces collected from a real hospital environment show that the system successfully tracks and distinguishes the mobile assets with 100% accuracy, and the system reduces about 97% of the asset management time.

Specifically, the contributions of the paper can be summarized in four-fold.

- This work develops a framework to classify failed nodes from mobile nodes, which can leave a monitoring region without failures using a sequence of neighbors of them. This method can be applied to many mobile asset-tracking services.
- This work proposes a node state classifier using trends extracted from the neighbor counts and the ratios of the boundary nodes included in the neighbor counts. To the extent of our knowledge, the proposed method is the first state classification technique, for mobile nodes, that does not use heartbeat messages or routing protocols.
- This work classifies a mobile node disconnected from a wireless sensor network as either a faulty node within a monitoring region or a node that leaves (or already left) the region without failures based on the trends of connectivity metrics.
- This work presents simulations and experiments using a network simulator, and real datasets with the proposed classifier are used. Their results indicate that this state classifier can detect most failed nodes that cannot be identified properly by conventional methods.

The rest of this paper is organized as follows. The related work and background of this study are presented in Section 2. The architecture of the mobile asset tracking system and its components are presented in Section 3. The failure detection method is explained in Section 4. The experimental environment and the experiment results are presented in Section 5. Finally, conclusions are given in Section 6.

## 2. Related Work

Several works on node failure detection in WSNs are briefly reviewed in this section.

Meier *et al*. [20] and Rost and Balakrishnan [21] proposed a distributed node monitoring tool (DiMo) and Memento, respectively. DiMo and Memento are network management tools for WSNs. These methods divide all nodes into two categories: observer nodes and remote nodes. Each remote node transmits heartbeat messages to its observer node periodically. If the observer node does not hear any heartbeat message for a certain threshold from the remote node, the observer considers the remote node as a faulty node. However, they do not consider a situation in which a mobile node can leave a WSN without failures.

Zia *et al*. [22] proposed a node failure detector that distinguishes between a node failure and a node movement. This method uses two types of nodes: observer nodes and target nodes. When a target node leaves the communication range of an observer node, the observer cannot distinguish between the target’s movement and the target’s failure. In order to handle this situation, the observer uses additional information obtained from one of the initiated nodes in the network. Initiated nodes are a few designated nodes that collect neighbor information from all nodes by propagating a message periodically. When an initiate node receives a message including “the target is alive” from one of the neighbors of the target, the initiator relays the response to the observer. The observer then recognizes that the target has moved. However, the method also does not consider a situation in which the target can leave the network.

Ramanathan *et al*. [23] proposed Sympathy, a tool to debug and detect failures occurring in WSNs. Sympathy runs at individual node, and failure detection occurs at a sink node. There are two types of packets in WSNs: monitored traffic and metric traffic. Each sensor node produces the former, and Sympathy produces the latter. Sympathy monitors the traffic to identify failure nodes and to decide the sources of failures. If a node produces monitored traffic less than a given threshold, Sympathy considers the node to be failed. However, Sympathy is not applicable to mobile nodes that leave the monitoring area without failures. As the sink node cannot hear any messages from all mobile nodes that normally leave the WSN, Sympathy incorrectly considers them to be failed.

Kim and Chung [24] proposed a failure detection method based on the connection state of a mobile node and its battery lifetime. It is used to detect faulty mobile nodes in mobile asset tracking systems. This method runs on a central server. A mobile node is attached to a mobile object, which is mobile within a monitoring region, leaves it freely and then returns to the area repeatedly. Therefore, the status of a mobile node is either in or out. This failure detection method using the connection state is primarily intended for detecting failed nodes with the in status; on the other hand, the method using the battery lifetime considers the out status. Although a battery lifetime estimator can detect failed nodes that have the out status and the low battery power at the same time, it cannot detect failed nodes that have sufficient battery power. On the other hand, the proposed method in this paper detects failed nodes with the out status regardless of the battery level.

Duche and Sarwade [25] proposed a node failure detection method using the the round-trip delay time occurring in both directions along a routing path in WSNs. This method detects failure nodes by measuring the latency in a round-trip path and comparing it to a certain threshold. The round-trip delay time of a failed node will be higher than the threshold value or infinite. However, this method cannot detect the failure of a mobile node that can leave the network without failure.

In summary, previous methods can efficiently detect node failures in certain ways. As they use a connection state determined by using heartbeat messages, those methods consider all mobile nodes with the left state to be a failed node. However, most of those methods do not consider a situation in which a mobile node can leave the monitoring region. Therefore, those methods erroneously consider a mobile node that has left the monitoring region without failures to be a failed node. To overcome this shortcoming, the classification method presented in this work distinguishes a faulty node from mobile nodes, which normally disconnects from the wireless sensor network.

## 3. System Component

### 3.1. Description

**Figure 2:**
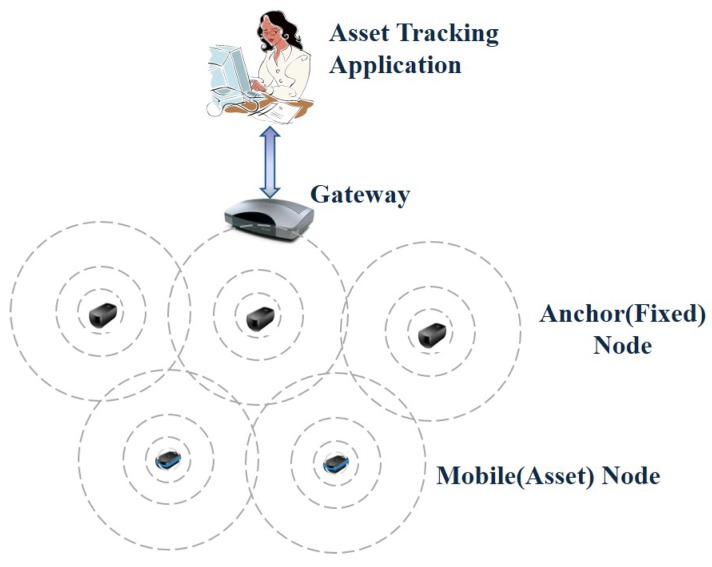
System architecture [24].

This section formalizes the system architecture of a medical asset tracking system developed in this work and the features of each component composing the system. The system architecture is shown in Figure 2. This system is composed of four-tuple S=(T,G,A,M), where *T* is the asset tracking application, *G* is the gateway, $A=a_{1},a_{2},...,a_{m}$ is the set of anchor nodes and $M=m_{1},m_{2},...,m_{n}$ is the set of mobile nodes [24].

A wireless sensor network is developed based on the ZigBee specifications and on IEEE 802.15.4 [26]. The devices included in the network form a multiple-depth tree by themselves, exchanging messages with one another. They operate under the beacon-enabled mode to achieve a low duty cycle. A personal area network coordinator (PANC) included in the gateway starts the network formation process, and each node maintains a neighbor list table. This table typically contains neighbors’ addresses and received signal strength indication (RSSI) values for each connection. The anchor nodes and the mobile nodes are developed on the same hardware platform with the same software running on them. However, their functions are different with respect to their device type.

A mobile node is attached to a medical asset and always becomes a leaf node of the network. This node is a battery-powered device with a battery-saving sleep mode. If an anchor node and a mobile node can exchange radio signals, then the anchor node becomes a neighbor of the mobile node. However, a mobile node does not include other mobile nodes as a neighbor. Initially, each mobile node selects a parent node from neighbors that send signals that exceed a threshold. If the signal strength from the current parent is less than the threshold, the mobile node selects another parent node. Each mobile node wakes up periodically and exchanges polling messages with its parent to check whether the wireless connection between them is valid. After confirming the connection, this mobile node gathers RSSI values from its neighboring anchor nodes and sends them to its parent. After transmitting the data, the node enters a sleeping phase.

Anchor nodes are stationary nodes at a fixed location that form a tree network structure to transmit sensing data generated by each node to a gateway. They are installed in a monitoring region or on the boundaries of the target monitoring area and provide a high spatial resolution and a monitoring infrastructure for the mobile nodes. Every anchor node already knows its own position and acts as a router, which forwards messages for mobile nodes and other anchor nodes toward and from the gateway. Each anchor node uses heartbeat messages to detect disconnections of its neighbors.

The gateway is considered as an interface between the application and the wireless sensor network. The gateway receives RSSI values reported by a mobile node through the anchor nodes. The trilateration algorithm in the gateway determines the location of the mobile node using RSSI values received from the node. This location algorithm also uses filters to eliminate invalid RSSI values and to increase the location accuracy. This filter is a simple threshold-based algorithm. This location algorithm will not be discussed in detail in this paper as the details are outside the scope of this study.

The asset tracking application system gathers the location data of all individual nodes from the gateway. It takes the role of keeping track of the current locations of all mobile nodes, determining the enter/exit state of each mobile node, counting the number of assets within (and outside) the monitoring area and displaying all of the gathered data on its graphical user interface.

### 3.2. Testbed

**Figure 3:**
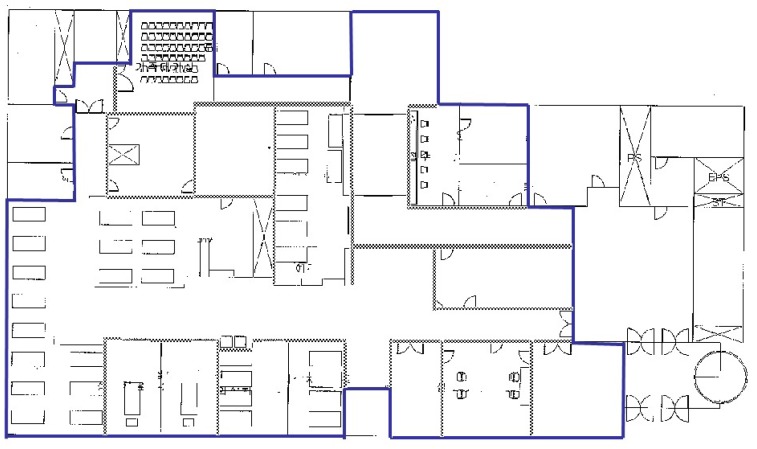
Map of the emergency room.

The 40 m × 30 m emergency room where the prototype system was installed is enclosed with entrances and concrete walls. There are four treatment sections, all of which have no doors and are partitioned by concrete walls, a computed tomography (CT) room, two X-ray rooms, a control room for CT and X-ray scans, nurses’ and doctors’ offices, a waiting room, a staff station, a storage room and a hallway. Concrete walls and metal doors also partition the control room, CT room and X-ray rooms. The emergency room has about 100 medical assets, and four nurses spend about 30 min manually checking whether each asset is within the target area. Nurses selected 21 mobile medical objects, a subset of the ∼100 assets, to evaluate the developed system. The medical asset tracking system is composed of an application, a gateway, 38 anchor nodes deployed within the emergency room or on the boundary of the area and 21 mobile nodes attached to 21 mobile medical objects, one for each object. The medical assets here are two wheelchairs, four ventilators, five syringe pumps and 10 IV poles. Using the system, one nurse can finish the work within one minute. Figure 3 shows a map of the emergency room enclosed with bold solid lines. They indicate the boundary of the monitoring area.

From an operational perspective, the system required maintenance approximately once every two weeks to exchange the batteries on the mobile assets. Each mobile asset was randomly mobile with a wakeup-and-report interval of 30 s. The RSSI-based location estimations showed an accuracy of ∼2.5 m, which was enough to satisfy the staff’s requirements in a non-line-of-sight (NLOS)-dominated environment. On the other hand, the anchor nodes were powered using wall plugs; thus, they were awake for the entire testing period.

## 4. Node Classification

In this section, a node classification method is introduced. The classification is between nodes that failed, therefore not being able to transmit RSSI-included messages, and nodes that leave the target detection region. These nodes are defined as failed nodes and left nodes, respectively, and comprehensively termed disconnected nodes. A straightforward solution is to use the last reported location of a disconnected node. If the final location of a disconnected node is within a monitoring area, then the node is considered to be a failed node. Otherwise, the node is considered to be a left node, which naturally left the target monitoring area. However, this method is not suitable for this system, since all anchor nodes are deployed within the monitoring area or on the boundary of the region. As a result, the developed system and many monitoring systems are incapable of “detecting” packets that are external to this area.

**Figure 4:**
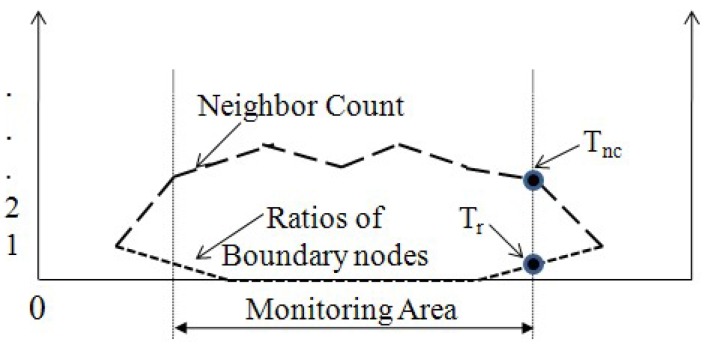
Concepts on the trends of the neighbor counts and the ratios of the boundary nodes and two thresholds.

To overcome this shortcoming, the method proposed in this work uses two trends associated with the neighbor counts and the ratios of the boundary nodes included in the neighbors. Let *m* be a mobile node, *B* be the boundary of a monitoring area, *d* be the Euclidean distance between *m* and *B*, N(m) be the number of *m*’s neighbors and $N(B_{m})$ be the number of $m^{\prime }s$ neighbors on *B*, $N\left(B_{m}\right)\leq N\left(m\right)$. Then, while *m* moves far away from the monitoring area, as *d* increases, N(m) decreases and $N\left(B_{m}\right)/N\left(m\right)$ increases. Finally, both $N(B_{m})$ and N(m) become zero when *m* loses all communication links with its neighbors. Figure 4 shows these concepts.

In Figure 4, the thresholds, $T_{nc}$ and $T_{r}$, are used to determine whether a disconnected mobile node sends its last value from either inside or outside of the monitoring region. To calculate $T_{nc}$ and $T_{r}$, a mobile node disconnected from a WSN is mapped onto the nearest location on the boundary of the monitoring area from its last location. Then, the two values, $T_{nc}$ and $T_{r}$, are estimated. Based on the trends and the thresholds, the proposed method classifies a disconnected mobile node as either a left node or a failed node. The two trends are complementary to each other in various environments. For example, the trend of the neighbor counts reported by a mobile node within the monitoring area may decrease due to barriers, such as walls, other medical assets and people. In this case, an incorrect decision caused by the trend of the neighbor counts can be corrected by observing the ratios of boundary nodes. However, the proposed method does not incur an additional communication cost, because the neighbors’ data are essential to calculate the position of a mobile node in a radio-frequency-based positioning system.

From the above concepts, the proposed method can possess the following properties. Let $A_{I}$ and $A_{O}$ be the area within (*i.e.*, inside) and outside the region of a monitoring area, in which the radio signals of the boundary nodes can propagate, respectively. If the ratio of the boundary nodes of a mobile node is greater than zero, the node is considered to be outside the monitoring area with probability $A_{O}$/($A_{I}+A_{O}$), where $A_{O}\cap A_{I}=\phi$. In addition, let *m* be a mobile node that has left a sensor network without failures and stayed outside a monitoring area, *l* be the line segment crossing *v* and *w*, which are *m*’s two nearest nodes on the boundary of the area, $d_{1}$ be the length of *l* and $d_{2}$ be the shortest distance between *l* and *m*. If $0<d_{1}\leq r$, the communication range of a node, and *m* is located in the line segment, *l*, with the distance $d_{2}>0$, then $d_{2}$ is greater than $\sqrt{r^{2}-{\left(d_{1}/2\right)}^{2}}$.

### 4.1. Data Sequence

When a mobile node is disconnected from a WSN, two sequences are extracted from the history of its neighbor counts. The neighbor counts of a mobile node are a set of observations $P_{t}$, each of which is recorded at time *t*, because they are reported continually at every update period. Therefore, the history of neighbor counts is represented as the time series $P=P_{1},P_{2},\cdots ,P_{z-1},P_{z}$, where $P_{z}$ is the last value reported by a mobile node. Each $P_{i}$ has the property, $1\leq P_{i}\leq \left|A\right|$, where *A* is the set of anchor nodes and 1≤i≤z. After the time *z*, the mobile node is disconnected from the network. As the history contains a large number of values, it is not feasible to extract the trends from the entire history *P* given the resource limitations on a sensor node platform. Therefore, a short sequence *Q* is extracted from *P*. To select *Q*, a data window expands from $P_{z-1}$ of the history *P* of a disconnected mobile node to the first value that is greater than $T_{nc}$. *Q* is represented as $Q=Q_{1},Q_{2},\cdots ,Q_{w-1},Q_{w}$, where $Q_{1}=P_{i},\cdots ,Q_{w-1}=P_{z-1},Q_{w}=P_{z}$, where |Q|≤|P|,i=z−w+1. Another sequence *R* is extracted from *Q*, and it is represented as $R=R_{1},R_{2},\cdots ,R_{w-1},R_{w}$, where $R_{j}=B_{j}/Q_{j},|R|=|Q|,0\leq B_{j}\leq Q_{j},1\leq j\leq w$ and $B_{j}$ indicates the number of boundary nodes contained in $Q_{j}$.

### 4.2. Estimating Neighbor Counts

The number of neighbors of a mobile node at a location within a monitoring area or on the boundary of the area is estimated in this section. The estimated neighbor count is used to determine $T_{nc}$ and $T_{r}$ and to calculate the missing neighbor rate, defined as $\left(NC_{E}-NC_{O}\right)/NC_{E}$, where $NC_{O}\leq NC_{E}$, and $NC_{O}$ and $NC_{E}$ indicate the observed neighbor count and the estimated neighbor count, respectively.

#### 4.2.1. Grid Model

If it is assumed that anchor nodes are uniformly distributed in a grid pattern, then the number of neighbors of a mobile node is estimated as follows. Let $X_{1},X_{2},\cdots ,X_{n}$ be the number of neighbor nodes that a mobile node can have when they are measured with a fixed interval. As a sensor network contains *N* anchor nodes, the probability that the radio coverage of a mobile node contains *k* anchor nodes can be represented as P(X=k). Therefore, the probability that a mobile node has *k* neighbors is:

(1) P(X=k)=Nkpk(1−p)N−k

where *N* is the number of anchor nodes within the monitoring area and *p* is the probability that an anchor node falls within the mobile node’s radio coverage.

It is assumed that anchor nodes are uniformly distributed within a rectangular monitoring area. The probability, *p*, is $A_{O}/A_{M}$, where $A_{O}$ represents the overlapping area between the radio coverage of a mobile node and the monitoring area and $A_{M}$ represents the area of the monitoring region.

The expected number of neighbor nodes of a mobile node is:

(2) $$\begin{array}{cc} E(X) & =\sum_{k=1}^{N}k\times P\left(X=k\right) \end{array}$$

(3) $$\begin{array}{cc} & =Np\times \sum_{k=1}^{N}\left(\frac{(N-1)!}{(N-k)!\times (k-1)!}\right)p^{\left(k-1\right)}{\left(1-p\right)}^{\left(N-k\right)} \end{array}$$

(4) $$\begin{array}{cc} & =Np\times 1=Np \end{array}$$

If the radio coverage of a mobile node contains the monitoring area, E(X) is equal to *N*. If the monitoring area contains the radio coverage or the two areas are overlapping, E(X) is equal to $\frac{\left(N\times \pi \times R^{2}\right)}{A_{M}}$, because $A_{O}$ is $\pi \times R^{2}$, where *R* indicates the radio range of a mobile node. If the two areas are overlapping, E(X) is equal to $\left(N\times A_{O}\right)/A_{M}$.

#### 4.2.2. Real Deployment

The grid model is a simple and computationally inexpensive method. However, it cannot be applied to this medical asset tracking system because the anchor nodes cannot be deployed in a grid pattern within the emergency room, which is not a rectangle, as well as it has several walls. In addition, anchor nodes were not allowed to be installed on a marble wall. Under these restricted circumstances, the number of neighbors of a mobile node is estimated in two phases, the filter phase and the refinement phase, to shorten the processing time. In the filter phase, the candidate neighbors of the node are found using a minimum bounding rectangle (MBR) in which the communication coverage of the node is contained. This phase is not computationally expensive because at most four comparisons are required to determine whether an anchor node is within the MBR. The candidate neighbors construct the set *C* defined as $C=c_{1},c_{2},\cdots ,c_{q}$, where C⊂A, the set of anchor nodes. Each element in *C* has the properties, $\forall c_{i}\in C$ and $c_{i}\in \left\{\left(x_{i},y_{i}\right)|\left(MinX\leq x_{i}\leq MaxX\right)\;and\left(MinY\leq y_{i}\leq MaxY\right)\right\}$, where $\left(x_{i},y_{i}\right)$ is the location of $c_{i}$, 1≤i≤q, and MinX,MinY,MaxX and MaxY are the coordinates of the MBR of a mobile node.

In the refinement phase, the candidate neighbors are checked for whether they are really the neighbors of the mobile node. The number of nodes processed in this phase is reduced due to the initial filter phase. To select the actual neighbors of the node from *C*, the distance between the node and $c_{i}$ in *C* is calculated. The actual neighbors of the node construct the set *N* defined as $N=n_{1},n_{2},\cdots ,n_{k}$, where N⊂C. Each element in *N* has the property $dist(m,n_{j})\leq r$, where dist is the distance function, *m* indicates the mobile node, $n_{j}$ indicates that of each element in *N*, 1≤j≤k and *r* is the communication range of mobile node *m*. Therefore, the number of neighbors of the mobile node equals |N|.

#### 4.2.3. Trend Detection

To extract the trend from *Q* and *R*, a linear regression model is used. This model finds the best-fitting straight line for all data in a data sequence and decides the trend of the sequence in accordance with the slope of the fitting line. The trend detection used here is the regression of neighbor counts over time and that of ratios of boundary nodes over time. Consider a regression model, where a dependent variable $y_{i}$ is linked to an independent variable $x_{i}$ through the following equation:

(5) $$y_{i}=ax_{i}+b$$

The equation above describes the relationship between *y* and *x* and represents a line with an intercept of *b* on the *y*-axis and a slope of *a*. The two values, *a* and *b*, for which the sum of the squares of the estimated errors is the minimum, have to be estimated. The estimation equation is given as follows:

(6) $$\text{Sum}\;\text{of}\;\text{Square}\;\text{Error}\left(SSE\right)=\sum_{i=1}^{n}{\left(ax_{i}+b-y_{i}\right)}^{2}$$

and:

(7) $$\frac{\partial SSE}{\partial a}=2\times \sum_{i=1}^{n}x_{i}\left(ax_{i}+b-y_{i}\right)$$

(8) $$\frac{\partial SSE}{\partial b}=2\times \sum_{i=1}^{n}\left(ax_{i}+b-y_{i}\right)$$

Setting these expressions equal to zero and solving for *a* and *b* produce:

(9) $$a=\frac{n\sum_{i=1}^{n}\left(x_{i}y_{i}\right)-\sum_{i=1}^{n}x_{i}\sum_{i=1}^{n}y_{i}}{D}$$

(10) $$b=\frac{\sum_{i=1}^{n}x_{i}^{2}\sum_{i=1}^{n}y_{i}-\sum_{i=1}^{n}x_{i}\sum_{i=1}^{n}\left(x_{i}\right)\left(y_{i}\right)}{D}$$

where $D=n\sum_{i=1}^{n}x_{i}^{2}-{\left(\sum_{i=1}^{n}x_{i}\right)}^{2}$; *a* represents the slope, and *b* represents the *y*-axis intercept. This slope represents the direction of the trend. A negative slope represents a decreasing trend; a positive slope represents an increasing trend; and zero represents that there is no change in the data sequence. Therefore, a left node from a monitoring area has a decreasing trend in *Q* and an increasing trend in *R* simultaneously.

### 4.3. Classification

The classification method proposed in this work uses a binary classifier that categorizes a mobile node disconnected from a WSN as either a failed node or a left node. This classifier uses four values. These are two trends extracted from *Q* and *R* and two thresholds $T_{nc}$ and $T_{r}$ used to determine whether a disconnected mobile node sends its last value from either inside or outside of a monitoring region. Therefore, the classification rule to distinguish between a failed node and a left node consists of four conditions related to the trends and the thresholds. The classification rule is given as:

(11) $$H=\left\{\begin{array}{cc} \text{Left}\;\text{Node}, & \text{if}\;S\left(Q\right)<0\;\text{and}\;Q_{w}<T_{nc}\;\text{and}\;S\left(R\right)>0\;\text{and}\;R_{w}>T_{r}\\ \text{Failed}\;\text{Node}, & otherwise \end{array}\right.$$

where *S* indicates the slope and $Q_{w}$ and $R_{w}$ indicate the last neighbor count and the last ratio of the boundary nodes, respectively, that a disconnected mobile node transmitted.

## 5. Performance Evaluation

To study the properties of the classification method presented in this work, several experiments are performed in two phases. In the first phase, the ns-2 simulator version 2.31 is used to capture the potential performance with different scenarios. In the second phase, real trace data are used, which were generated by the medical asset tracking system deployed in a real emergency room environment. This simulation evaluates the environmental changes, which affect the performance of the proposed classification scheme.

### 5.1. Simulation

#### 5.1.1. Environment

Two simulated networks are constructed for the simulations: one is for a grid model, and the other is for a real deployment. The simulated network for a real deployment is equal to that of the medical asset tracking system in Figure 3. A summary of the environment is shown in Table 1. Each mobile node wakes during every report interval, broadcasts a heartbeat message, collects responses from its neighbors and generates the neighbor data. In this simulation, 10 mobile nodes leave the monitoring area through a door, and 11 mobile nodes fail in the area. Although the communication range of actual nodes is 30 m in the free space, it is estimated to be reduced to about 12 m in the emergency room by the indoor path loss model defined by the International Telecommunication Union (ITU) [27].

**Figure 5:**
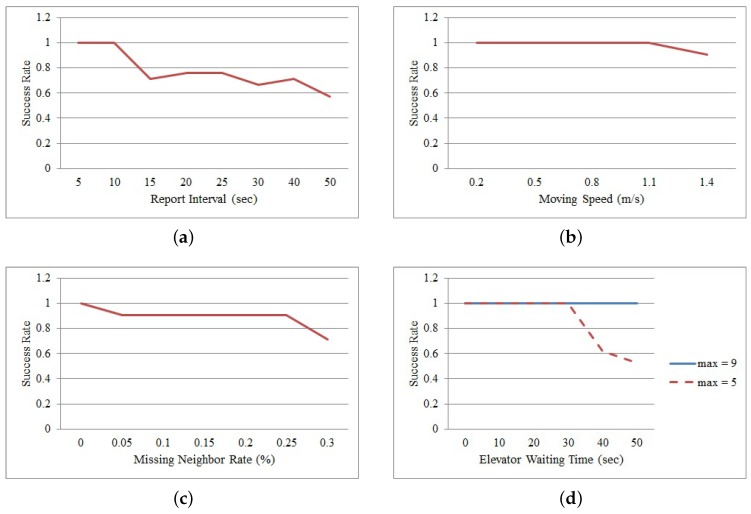
Detection results of both failed and left nodes in a grid model according to the parameter changes. (**a**) Report interval; (**b**) Moving speed; (**c**) Missing neighbor rate; (**d**) Elevator waiting time.

**Figure 6:**
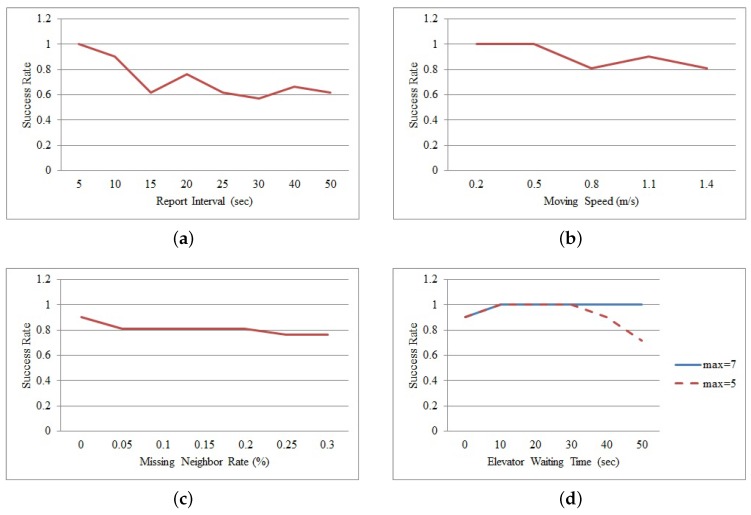
Detection results of both failed and left nodes in a real deployment model according to the parameter changes. (**a**) Report interval; (**b**) Moving speed; (**c**) Missing neighbor rate; (**d**) Elevator waiting time.

The performance of the proposed method is compared in terms of the report interval, moving speed, missing neighbor rate and elevator waiting time. The report interval indicates the period of time during which a mobile node collects and sends its neighbor data to the sink node. In this simulation, the moving speed of a mobile node is assumed to be less than or equal to 1.1 m/s because the node attached to a mobile medical asset in an emergency room moves slowly with a patient. It is slower than the range of normal walking speeds, 1.2∼1.4 m/s, of a person; however, it is faster than the walking speeds, 0.6 m/s, of hospitalized patients [28]. The missing neighbor rate is affected by data collisions. If the number of collisions increases, the missing neighbor rate increases, and the number of neighbors of a mobile node decreases. The elevator waiting time is the time required to take an elevator when a mobile node goes to another department. Figure 5 and Figure 6 show the results of this experiment. In the figures, the metric, SR(successrate), is defined as SR=(FF+LL)/(F+L), where FF and LL indicate the numbers of failed nodes and left nodes, which are determined by the proposed method, respectively; *F* indicates the number of failed nodes and is set to 11; and *L* indicates that of left nodes and is set to 10.

#### 5.1.2. Report Interval

This section evaluates the impact of the time interval in which a mobile node reports its values. The report interval varies from 5 to 50 s. The moving speed, the missing neighbor rate and the elevator waiting time are set to 1.1 m/s, 0% and 0 s, respectively. Figure 5a and Figure 6a show the results. The figures show that the success rate decreases when the report interval is greater than 10 s. These results indicate that some left nodes are classified as a failed node incorrectly because they reported their last values within the monitoring area. Therefore, most of the left nodes with an incorrect state do not satisfy the condition $Q_{w}<T_{nc}$. On the other hand, all failed nodes within the area are correctly considered as failed nodes, and they are divided into three groups. First, the failed nodes, which move within the area, do not commonly satisfy the two conditions of $Q_{w}<T_{nc}$ and $R_{w}>T_{r}$ in the classification rule. Second, the failed nodes that are continually located near the door do not commonly satisfy the two conditions S(Q)<0 and S(R)>0. Finally, the failed nodes, which are continually located far from the door, do not satisfy the four conditions. However, the proposed method correctly classifies all failed nodes and all left nodes when the report interval is less than or equal to 5 s. In this case, the moving distance of a mobile node between two consecutive reports is less than the communication range. Therefore, the left nodes can report their last values from outside of the monitoring area at least once before being disconnected from the sensor network, and they satisfy the classification rule.

#### 5.1.3. Moving Speed

This section evaluates the impact of the moving speed of a mobile node. The moving speed varies from 0.2 to 1.4 m/s. The report interval, the neighbor missing rate and the elevator waiting time are set to 10 s, 0% and 0 s, respectively. Figure 5b and Figure 6b show the results. The figures clearly show that the success rate decreases when the moving speed is increasing. These results indicate that several nodes are classified incorrectly as failed nodes within the monitoring area because they reported their last values within the area. Some nodes do not satisfy the condition $Q_{w}<T_{nc}$; others do not satisfy the condition $R_{w}>T_{r}$. However, the proposed method correctly classifies all failed nodes and all left nodes when the moving speed is less than or equal to 0.5 m/s. In this case, the moving distance of a mobile node between two consecutive reports is less than the communication range. This means that the left nodes can report their last values from the outside of the monitoring area at least once before they are disconnected from the sensor network. Therefore, all left nodes are classified correctly.

#### 5.1.4. Missing Neighbor Rate

This section evaluates the impact of the missing node rate. In this simulation, the missing neighbor rate varies from 0% to 0.3%. The report interval, the moving speed and the elevator waiting time are set to 10 s, 1.1 m/s and 0 s, respectively. The missing neighbor rate is used to calculate the threshold, $T_{nc}$, which distinguishes between an inside node and an outside node. The average value of the missing neighbor rates observed at all mobile nodes is 0.11%, and the range of the missing neighbor rates is 0%∼3.33%. Figure 5c and Figure 6c show the results. As shown clearly in the figures, the success rate decreases when the rate is greater than or equal to 0.05%. These results indicate that some left nodes are classified incorrectly as failed nodes. The reduced threshold, $T_{nc}$, is the main cause of these incorrect decisions. The classification condition related to the rate is $Q_{w}<T_{nc}$, and the threshold is calculated as $T_{nc}=T_{nc}\times \left(1-missing\;neighbor\;rate\right)$. As $T_{nc}$ decreases and $Q_{w}$ does not change, the number of failed nodes that do not satisfy the condition $Q_{w}<T_{nc}$ increases. The proposed method has a highest success rate when the missing neighbor rate equals zero. As collisions among data transmissions can affect nodes in a non-uniform way, the most suitable value for the missing neighbor rate is zero, which can be applied to all nodes in common without incorrect decisions.

#### 5.1.5. Elevator Waiting Time

This section evaluates the impact of the waiting time to get on an elevator. As the emergency room is in a 12-story building with two elevators and the elevators are located outside the emergency room, a patient with a mobile medical asset has to take an elevator to go to another department. Therefore, the waiting time for an elevator makes a mobile node send its value frequently from outside of the monitoring area. In this simulation, the report interval, the moving speed and the missing neighbor rate are set to 10 s, 1.1 m/s and 0%, respectively. The elevator waiting time varies from 0 to 50 s. Figure 5d and Figure 6d show the results. The “max” in the figures indicates the maximum window size.

In Figure 5d and Figure 6d, the graphs (max = 5) clearly show that the success rate decreases when the waiting time is both 40 and 50 s. This result indicates that some left nodes are classified incorrectly as failed nodes. These incorrect decisions occur because *Q* and *R* have the same data. If the waiting time is long, a mobile node sends many reports at the same location outside of the monitoring area. In this case, *Q* and *R* can contain the same neighbor data if the node sends the same value more than five times, the maximum length of *Q* and *R*. As these identical values in *Q* and *R* generate an unchanged trend, the left node is considered as a failed node. In this case, the range of the lengths of *Q* and *R* is 2∼5.

This problem can be solved if the lengths of *Q* and *R* are variable. The length of *Q* increases until a value greater than the threshold $T_{nc}$ appears. Then, *Q* and *R* can contain at least one value reported by a mobile node in the monitoring area. The graphs (max = 7 and max = 8) in the figures show the result using the variable length. The range of the lengths is 2∼9 in Figure 5d, and it is 2∼7 in Figure 6d. The figures show that the proposed classifier separates all failed nodes from all left nodes correctly.

### 5.2. Evaluation with Real Datasets

Unlike simulation-based evaluations, the environmental changes, which affect the performance of the proposed method, become complicated when operating in real environments. To test the proposed algorithms in such systems, operational traces from the initial system deployed at the hospital are collected. This dataset consisted of the reports written by the nurses and the log data generated by the system for a two-week duration. Nurses in the emergency room counted the number of mobile assets outside and inside the detection region three times a day. The nurses also compared their manual counts with the system and check if the two results differ. If the results do differ, the nurses record the numbers and the names of the assets with incorrect statuses in their reports. Of the 14-day experimental period, four different days with the differences in the nurses data and system data were observed. The data from these days were selected as the experimental data for the real dataset-based evaluations.

The performance of the proposed method is compared to those of two other previously proposed schemes: the observation of heartbeat messages and the estimation of battery lifetime [19]. The observation that is similar to the method proposed in [17] is used as the baseline approach. An anchor node in the observation monitors heartbeat messages that a mobile node sends periodically. The anchor node is one of the neighbors of the mobile node. If an anchor node does not receive the message within 3 min from a mobile node, it sends a message indicating that the “mobile node leaves” the gateway. The gateway considers the mobile node to have left the detection region if it does not receive a message indicating a network-joining message within 2 min from any anchor node in the network. The estimation of battery lifetime estimates whether a mobile node has low battery power. This method considers a mobile node as a failed node if the node with a low battery level is disconnected from the WSN. To evaluate the performance levels of these methods, the number of inside and outside nodes and their neighbor counts are extracted from the log data, which the system generated on the two aforementioned dates. In addition to the log data, the records containing the periods that indicate the times to replace the battery are collected. With these data, the three methods detect failure nodes from mobile nodes disconnected from the WSN. If a failure detection method identifies a failed node, it increases the number of inside assets and decreases the number of outside assets. The nurse’s reports are used as a reference to confirm whether the number of failure nodes detected by the methods is correct.

#### 5.2.1. Neighbor Count

**Figure 7:**
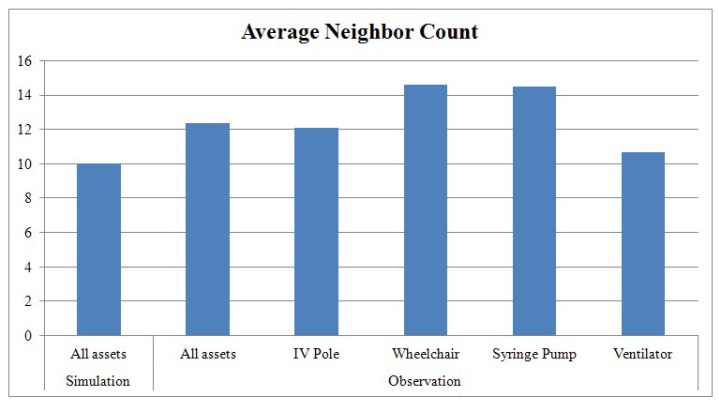
Average neighbor count in the simulation and in the emergency room.

The average neighbor counts of the mobile nodes are compared in this section They are collected both using simulation and real-world traces in Figure 7. In the emergency room, the average neighbor counts for different types of assets are slightly different given the fact that assets are stored in different places based on what they are. As Figure 7 clearly shows, the neighbor count of a mobile node in the emergency room is higher than that of a mobile node in the simulation. This is because several anchor nodes outside the communication range of a mobile node can communicate with the mobile node in the emergency room due to RF reflection.

#### 5.2.2. Detection Results of Inside and Outside Nodes

**Figure 8:**
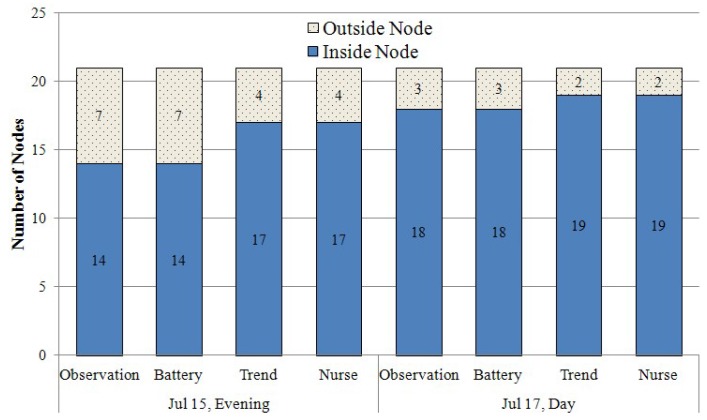
Detection results of the number of inside and outside nodes for real data generated in the emergency room [17]. (With the permission of IEEE publisher).

The numbers of internal and external node count computed for the three different methods are shown in Figure 8. Here, a two-day dataset is used to observe the performance in greater detail. The proposed method uses the variable lengths of both *Q* and *R*. The range of the lengths is 2∼10. In Figure 8, the first bar generated by the observation method indicates that 14 out of 21 nodes are inside the emergency room and that seven nodes are outside the area. This result is generated when the observation is applied to the log data generated by the mobile asset tracking system when the evening group used it on 15 July. The second bar on that date is generated by the battery estimation method. Its result is equal to that of the observation. However, their results are different from the counts in the fourth bar (written by the nurses on that date), since those methods cannot actively identify any failure nodes. Given that a mobile node can leave the emergency room, the observation incorrectly considers the mobile nodes, which fail while within the emergency room as nodes that left the monitoring area (*i.e.*, left nodes). The battery lifetime estimation method also incorrectly considers the failed nodes with sufficient battery power as left nodes. On the other hand, the proposed method using trend analysis successfully detects the three failed nodes and four left nodes and then changes the number of inside and outside assets (the third bar on that date). Although a mobile node reports its data every 30 s, four left nodes report their data at least once when outside the monitoring area while they wait for an elevator before moving to a different department. This additional report from an outsider node (*i.e.*, left node) allows the system to correctly classify all left nodes properly. Unlike these nodes, the three failed nodes do not commonly satisfy the condition $Q_{w}<T_{nc}$, because they failed inside the monitoring area (*i.e.*, emergency room). The classification result from the proposed scheme successfully matched the observations made manually by the nurses; thus, this result indicates that the classification process was successful. The result for 17 July is also similar to that of the observations for the 15 July data. By observing the results generated by the proposed method and the nurse’s reports, it is clear that the classifier proposed in this work identifies all of the failed nodes that the other approaches cannot identify.

### 5.3. Discussion

Throughout this evaluation, the performance of the method presented in this work is evaluated in terms of the report interval, moving speed, missing neighbor rate and elevator waiting time under the environments: (1) the anchor nodes are installed both on the boundary of the monitoring area and within it; and (2) a mobile node can move far away from the area without any failures. From the evaluation results, it is clearly understood that the classification between a left node and a failed node is difficult by the fact that it cannot be assured that a mobile node will report its values outside the monitoring area before it is entirely disconnected from the wireless infrastructure (of anchor nodes). The mobile medical asset tracking system can adjust the report interval to increase the classification rate between a failed node and a left node. It is clear that the classification can be near 100% when the report interval is less than 10 s in Figure 5a and Figure 6a. However, a short report interval shortens the lifetime of a mobile node due to frequent data transmissions. Attaching a tilt sensor [29] to a mobile node can solve this problem. Then, the mobile node with the tilt sensor can report its location and neighbor data when it moves continually for more than a predefined time. This predefined time can be configured with respect to the size of the monitoring area and a person’s average walking speed. If this scheme is adapted to a mobile node, the report interval of the node can be shorter (by being adaptive) and will only minimally impact the lifetime of the mobile nodes. When a mobile node reports its data every 30 s, its battery lifetime is about 14 days. A lithium battery is attached to each mobile node. The battery and node properties are shown in Table 2. On the other hand, the lifetime of a mobile node with a tilt sensor is estimated to be about 120 days under the two following assumptions. First, a mobile node exchanges a heartbeat with its neighbors every 120 s. Furthermore, if the mobile node recognizes that it consecutively moves for more than the predefined time (e.g., computed as 10 s in the target environment and a walking speed of 1.5 m/s), it assembles and reports the neighbor data. Second, the actual moving time of a mobile node, based on the empirically collected dataset, is estimated to be about 144 min, which is only 10% of an entire day. Therefore, by disabling reports while the mobile devices are stationary, the number of reports can be decreased and the lifetime of the nodes increased.

## 6. Conclusions

This paper proposes a classification scheme to distinguish nodes that failed to transmit packets (*i.e.*, failed nodes) and nodes that leave the monitoring area (*i.e.*, left nodes) under the environments, where mobile nodes can freely move around a monitoring region, leave the region and return to the area repeatedly. If a system to track mobile assets uses heartbeat messages to detect a left node without being aware of possible node failures, the system can generate errors of detecting nodes within the detection region as nodes that leave the area. The proposed method in this paper utilizes two trends that are complementary. One is extracted from a series of neighbor counts generated at each mobile node, and the other is extracted from the ratios of boundary nodes included in the neighbor counts. By using trends of these measurements, the proposed classification method successfully separates failed nodes from mobile nodes that disconnected from wireless sensor networks due to their mobility. The performance of the proposed scheme is compared to those of conventional schemes that use the heartbeat messages and the battery lifetime of mobile nodes. Performance evaluations using real datasets and an ns-2 simulator show that the proposed scheme can detect the mobile nodes that fail to provide proper performance and show that the proposed method has superior performance over conventional schemes. By properly detecting faulty nodes in wireless sensor networks, a system to track mobile assets can improve its reliability and increase its asset management performance. For future research, optimizations for power consumption will be studied to prolong the lifetime of each mobile node. This issue will consider various techniques, such as communication energy consumption, low power sensing devices and small antennas.

## Figures and Tables

**Table 1:** Summary of simulation environment.

Parameter	Value
Area	40 m × 30 m
Number of anchor nodes (grid pattern)	36
Number of anchor nodes (real deployment)	38
Number of mobile nodes	21
Communication range	12 m
Average neighbor count (grid pattern)	7.95
Average neighbor count (real deployment)	10.04
Minimum length of both Q and R	2
Maximum length of both Q and R	5
Mobility model	Random Waypoint

**Table 2:** Summary of battery and node properties [24].

Parameter	Value
Capacity	1300 mAH
Duration of sleeping phase	30 s
Duration of polling phase	5 s
Duration of location updating phase	2 s
Sleeping current	0.07 mA
Polling current	12.5 mA
Location updating current	40 mA
